# Tomato Yellow Leaf Curl Virus Reprograms Polyamine Metabolism in *Bemisia tabaci* MED to Enhance Viral DNA Accumulation

**DOI:** 10.3390/molecules31111835

**Published:** 2026-05-26

**Authors:** Zitong Sang, Haolin Han, Fangfang Qi, Guoqiang Pan, Guanghui Zhang, Shaolong Qiu, Yan Wei, Zhenzhen Zhang, Hengjia Zhang, Jinxing Xia

**Affiliations:** State Key Laboratory of Agricultural and Forestry Biosecurity, MOA Key Lab of Pest Monitoring and Green Management, College of Plant Protection, China Agricultural University, Beijing 100193, China; 18846914264@163.com (Z.S.); hanhaolin100@163.com (H.H.); qff1505481105@163.com (F.Q.); pgq341703@163.com (G.P.); zgh13949457565@163.com (G.Z.); s20233193332@cau.edu.cn (S.Q.); weiyan960619@163.com (Y.W.); zhangzhenzhen169@163.com (Z.Z.); hengjiazhang@nwafu.edu.cn (H.Z.)

**Keywords:** TYLCV, polyamines, *Bemisia tabaci*, insect vector, immune

## Abstract

Tomato yellow leaf curl virus (TYLCV) is a major plant pathogen that spreads worldwide through persistent circulative transmission by *Bemisia tabaci*. During transmission, TYLCV crosses several physiological barriers in the insect vector, evading immune defenses and altering host metabolic pathways to facilitate viral accumulation. Polyamines, essential for maintaining nucleic acid stability and promoting cellular processes, are known to play a critical role in viral accumulation. However, their role in TYLCV accumulation within *B. tabaci* is not well understood. Here, we demonstrate that TYLCV infection leads to significant alterations in polyamine levels in *B. tabaci*, with polyamine availability positively affecting viral DNA accumulation. Polyamine availability leads to higher viral loads and suppresses the expression of immune and MAPK signaling genes. These findings provide new insights into virus–vector and metabolic interactions underlying viral persistence in insect vectors.

## 1. Introduction

*Bemisia tabaci* (Gennadius) is a major pest that damages agricultural crops worldwide [1,2,3]. It directly feeds on plants and also acts as a vector for over 400 plant viruses, including *Begomovirus*, *Crinivirus*, *Ipomovirus*, and so on [4,5,6,7]. Additionally, its secretion of honeydew reduces plant photosynthesis and growth, causing yield losses ranging from 20% to 100% [3]. Tomato yellow leaf curl virus (TYLCV; genus *Begomovirus*, family *Geminiviridae*) is one of the most damaging constraints on tomato production worldwide, largely because epidemics track the rapid spread and high vector competence of the *B. tabaci* [8,9,10].

In the field, TYLCV is transmitted persistently in a circulative manner [10,11]. In this transmission mode, TYLCV virions ingested with phloem sap must cross the gut epithelium, circulate within the hemolymph, and ultimately enter the primary salivary glands before transmission to a new host plant [12,13,14]. TYLCV is capable of replicating within its whitefly vector *B. tabaci*, thereby ensuring persistent virus transmission [15,16]. This process is not passive but is shaped by coordinated interactions between the virus and the physiological state of the vector [11,14]. Such a transmission strategy imposes stringent requirements on viral particles, which must remain virion-stable and biologically competent while persisting within the internal environment of the insect vector [16,17,18]. Under such constraints, increasing evidence across virus host systems indicates that viral infection is frequently accompanied by endogenous metabolic reprogramming of the host, reflecting host defensive responses and virus-associated adaptations that can shape the physiological context of virus–vector interactions [19,20,21,22].

Polyamines are small, ubiquitous aliphatic polycations, primarily putrescine, spermidine, and spermine [23]. Owing to their positive charges, polyamines readily interact with negatively charged biomolecules such as DNA, RNA, and phospholipids, thereby playing essential roles in nucleic acid stabilization, chromatin organization, transcription, translation, and cell proliferation [24,25]. Beyond these fundamental cellular functions, polyamines have emerged as critical host factors exploited by viruses during infection. Accumulating evidence indicates that polyamines are required for efficient viral replication across diverse virus families, acting at multiple stages of the viral life cycle, including genome replication, gene expression, and virion assembly [26,27,28]. For DNA viruses, polyamines are frequently associated with virions, where they neutralize the negative charge of viral DNA, promote genome compaction, and enhance genome stability within the capsid [29,30]. Consistent with these roles, pharmacological depletion of cellular polyamines markedly restricts DNA virus replication and reduces viral DNA accumulation [31,32].

In insect-borne plant virus systems, polyamine-centered metabolic rewiring has been linked to enhanced viral infection or virion assembly in hemipteran vectors [33,34]. However, it remains unclear whether *B. tabaci* exhibits virus-specific patterns of polyamine remodeling, particularly for viruses like TYLCV that require long-term persistence within the vector’s internal environment. Here, we show that TYLCV infection is associated with pronounced remodeling of host polyamine pools. In contrast, tomato chlorosis virus (ToCV), which is transmitted by *B. tabaci* in a non-persistent manner, does not induce dynamic changes in polyamine levels. Additionally, the availability of exogenous polyamines can directly modulate TYLCV DNA accumulation in whiteflies, a relationship that is consistent with potential direct effects of polyamines on viral genome stability or replication efficiency. These findings suggest that polyamine levels are positively correlated with viral accumulation, with polyamines enhancing viral load and subsequently inhibiting key antiviral immune pathways and MAPK signaling.

## 2. Materials and Methods

### 2.1. Whitefly Colony and Plant Material

A virus-free colony of *B. tabaci* Mediterranean species (MED) was maintained on healthy tomato (*Solanum lycopersicum* Zhongza No. 9) in insect-proof cages at 25 ± 1 °C, 60–70% relative humidity, and a 14:10 h (light:dark) photoperiod.Adult whitefly 2–7 days post-emergence were used. Unless stated otherwise, each biological replication consisted of a pooled cohort of 30–100 adults collected from independent cages/plant sets.

### 2.2. Plants and Early Acquisition Time

TYLCV-infected tomato source plants were maintained separately from virus-free plants. Source plant infection was verified before each experiment by TYLCV-specific PCR/qPCR targeting a *V1*/*CP* fragment. Adult whiteflies were allowed an acquisition access period (AAP) for 48 h on TYLCV-infected plants, after which adult whiteflies were collected immediately. Each sample consisted of 30 adult whiteflies with an approximately 1:1 female to male ratio, and each treatment included three independent biological replicates. Collected insects were snap-frozen in liquid nitrogen and stored at −80 °C.

### 2.3. Polyamine and DFMO Treatment

To supplement or deplete polyamines, putrescine, spermidine, spermine, and difluoromethylornithine (DFMO; eflornithine) were delivered by sucrose membrane feeding. Adults were allowed to acquire TYLCV for 48 h. After the acquisition period, adults were provided with 10% (*w*/*v*) sucrose containing polyamines at 0.01% or DFMO at 0.04% for an additional 48 h through a stretched parafilm membrane. Vehicle controls received 10% sucrose without polyamines or DFMO. Each sample consisted of 30 adult whiteflies with an approximately 1:1 female to male ratio, and each treatment included three independent biological replicates. Following polyamine or DFMO exposure, samples were immediately collected for DNA extraction. All standards were purchased from Sigma-Aldrich (St. Louis, MO, USA).

### 2.4. Polyamine Extraction

Frozen adult insect samples (10 mg) were homogenized on ice in 500 μL of 10% perchloric acid (PCA). 1,6-Diaminohexane was added as an internal standard at a constant concentration. The homogenates were incubated on ice for 1 h and then centrifuged at 15,000× *g* for 10 min at 4 °C. The resulting supernatants were collected and mixed with an equal volume of 2 M NaOH, followed by the addition of 10 μL benzoyl chloride. The reaction mixtures were incubated in a 48 °C water bath for 20 min to allow benzoylation of polyamines. Subsequently, 1 mL saturated sodium chloride solution was added, and the mixtures were vortexed thoroughly. Polyamines were extracted by adding 1 mL diethylether, followed by centrifugation at 5000× *g* for 10 min. The organic phase was collected and evaporated to dryness under a gentle stream of nitrogen. The residues were reconstituted in 100 μL of 80% methanol, and the resulting solutions were used for polyamine analysis. Perchloric acid, methanol, sodium hydroxide, diethyl ether, and sodium chloride were purchased from Sinopharm Chemical Reagent Co., Ltd. (Shanghai, China). 1,6-IS and benzoyl chloride were purchased from Aladdin Biochemical Technology Co., Ltd. (Shanghai, China).

### 2.5. Waters UPLC Polyamine Workflow and Quantification

Putrescine, spermidine, and spermine were quantified using a Waters ultra-performance liquid chromatography system coupled with tandem mass spectrometry (UPLC–MS/MS). Polyamines were analyzed following a standard UPLC workflow after benzoyl chloride derivatization. Briefly, derivatized samples were clarified prior to injection and subsequently analyzed without further modification. Chromatographic separation was performed on a C18 UPLC column (CSH C18, 2.1 × 50 mm, 3.5 μm) using a water/acetonitrile gradient containing 0.1% formic acid at a flow rate of 0.3 mL/min. Detection was performed in positive electrospray ionization mode under multiple reaction monitoring (MRM). Concentrations were calculated by internal-standard normalization. Polyamine abundances were reported as normalized amount per pooled sample. Formic acid was purchased from Thermo Fisher Scientific (Waltham, MA, USA), and acetonitrile was purchased from Sinopharm Chemical Reagent Co., Ltd. (Shanghai, China).

### 2.6. DNA Extraction and TYLCV DNA Quantification by qPCR

Total DNA was extracted from 30 adult whiteflies using a silica column-based insect DNA extraction workflow (TIANGEN Biotech Co., Ltd., Beijing, China). TYLCV DNA was quantified by SYBR Green (TIANGEN Biotech Co., Ltd., Beijing, China) using primers targeting a TYLCV *V1*/*CP* region. The whitefly reference gene *β*-*actin* was quantified in parallel from the same DNA preparations. Reactions included melt-curve analysis to confirm specificity. Relative TYLCV DNA abundance was calculated using the $2^{-∆\mathrm{Ct}}$ method. Unless otherwise stated, the 0 h group served as the control. Each biological replicate was run with three technical replicates.

### 2.7. RNA Extraction and RT-qPCR

Total RNA was extracted from 30 whitefly samples using TRIzol reagent (Vazyme Biotech Co., Ltd., Nanjing, China) according to the manufacturer’s recommendations. Agarose gel electrophoresis was used to determine the integrity of the RNA, and a NanoDrop 2000c spectrophotometer(Thermo Fisher Scientific, Waltham, MA, USA) was used to quantify the RNA. cDNA was synthesized using the PrimeScript II 1st strand cDNA synthesis kit (Takara Bio Inc., Kusatsu, Shiga, Japan) and PrimeScript RT kit (containing gDNA Eraser, Perfect Real Time) (TaKaRa) for PCR and RT-qPCR analysis, respectively. Host immune and MAPK marker transcripts were quantified by RT-qPCR (SYBR Green) and normalized to *β*-*actin* using $2^{-∆∆\mathrm{Ct}}$.

### 2.8. Effects of Knockdown of BtODC Expression by RNAi on the Polyamine Biosynthesis and Viral Infection in B. tabaci

RNA interference (RNAi) was performed to knock down *BtODC* expression in adult MED whiteflies by dsRNA feeding. Specific dsRNA primers targeting *BtODC* and *EGFP*, containing T7 promoter sequences at the 5′ ends, were designed using the SnapDragon tool (https://www.flyrnai.org/cgi-bin/RNAi_find_primers.pl, accessed on 2 March 2026) to minimize potential off-target effects (Table S1). Double-stranded RNA (dsRNA) was subsequently synthesized in vitro using the T7 RiboMAX Express RNAi System (Promega Corporation, Madison, WI, USA). RNAi assays were conducted using the whitefly feeding device described above. A feeding solution containing dsRNA (80 μL, 0.5 mg/mL dsRNA) was placed between two layers of stretched Parafilm membrane. For each treatment, 30 adult whiteflies were used per biological replicate, and each treatment consisted of three independent biological replicates. Adult whiteflies were allowed to feed on the dsRNA-containing diet for 48 h, after which RNAi efficiency was evaluated by qPCR. Whiteflies fed with dsRNA for 48 h were subsequently collected for polyamine quantification and viral DNA accumulation analysis. Each dsRNA treatment, including the dsEGFP feeding solution used as the control, consisted of three independent biological replicates.

### 2.9. Statistics

Data were presented as mean ± SEM of three independent biological replicates. Time-course data (0–96 h), exogenous polyamine supplementation data on viral DNA accumulation, and gene expression data were analyzed using one-way ANOVA (time × treatment/condition) followed by a multiple comparison test. Polyamine levels were analyzed using two-way ANOVA. All analyses were performed using the GraphPad Prism version 9.5.1.

## 3. Results

### 3.1. TYLCV DNA Accumulation Increases During the First 48 Post-Acquisition in MED Whiteflies

**Figure 1:**
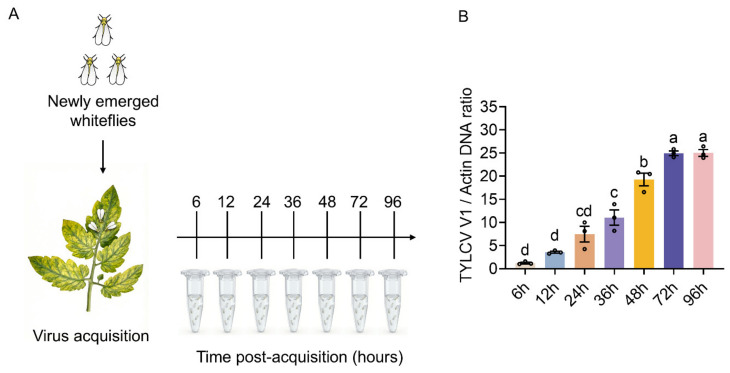
Temporal dynamics of virus DNA accumulation in *B. tabaci* following virus acquisition. (**A**) Schematic representation of TYLCV acquisition in newly emerged *B. tabaci* adults. (**B**) TYLCV DNA loads in MED whiteflies at indicated time points (6 h, 12 h, 24 h, 36 h, 48 h, 72 h, and 96 h) after acquisition feeding on TYLCV-infected tomato plants, as determined by qPCR and normalized to the *actin* gene. Data were presented as mean ± SEM from three independent biological replicates, with 30 whiteflies per replicate. Statistical significance was determined by one-way ANOVA followed by Tukey’s multiple comparison test. Different letters indicate significant differences among time points.

To define early within-vector dynamics, MED adults were collected at 6 h, 12 h, 24 h, 36 h, 48 h, 72 h, and 96 h, after acquisition on TYLCV-infected tomato plants (Figure 1A). TYLCV DNA abundance was quantified by qPCR and expressed as relative viral DNA normalized to *actin*. Across independent cohorts, TYLCV DNA accumulation increased over the first 48 h post-acquisition, with a continuous rise observed until 72 h, at which point viral DNA levels reached a plateau (Figure 1B), defining a reproducible early window in which viral DNA accumulation can be detected in the vector.

### 3.2. TYLCV Infection Induces Dynamic Changes in Vector Polyamine Pools

**Figure 2:**
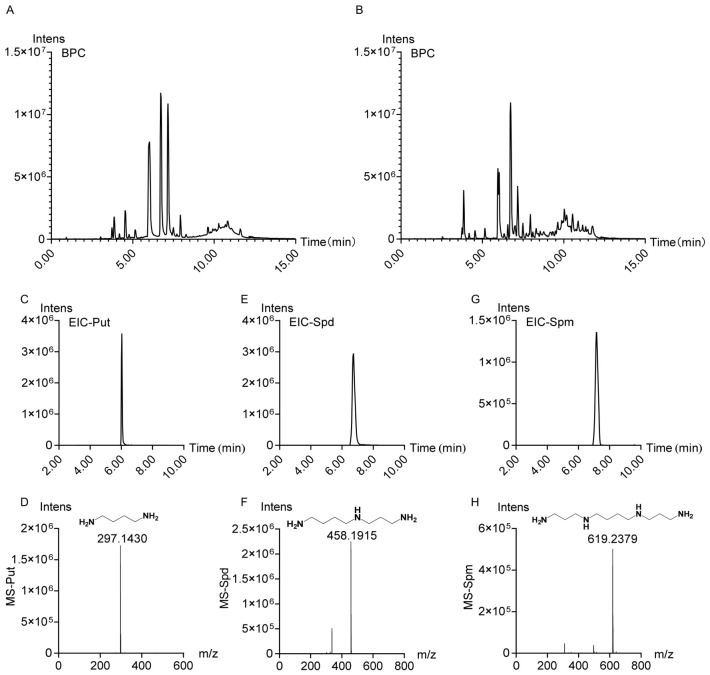
Polyamine detection in *B. tabaci* using UPLC-MS/MS. (**A**) Base peak chromatogram (BPC) of a standard polyamine mixture. (**B**) Base peak chromatogram (BPC) from whole body of adult *B. tabaci*. The separation of derivatization polyamines in an extraction within 5–10 min. (**C**–**H**) Extracted ion chromatogram (EIC) and MS spectra obtained from the whole body of adult *B. tabaci*.

To establish polyamines as a quantifiable and experimentally metabolic system in *B. tabaci*, we first characterized the composition and relative abundance of endogenous polyamines in adult whiteflies using an ultra-performance liquid chromatography–mass spectrometry (UPLC-MS/MS) workflow. Three canonical polyamines, putrescine (Put), spermidine (Spd), and spermine (Spm), were consistently detected in whole body extracts, each exhibiting distinct retention times and characteristic fragment ion spectra that matched those of authentic standards (Figure 2). Extracted ion chromatograms showed well-resolved peaks with high signal-to-noise ratios and consistent peak shapes across independent biological replicates, enabling reliable integration and relative comparison. Together, these data demonstrate that polyamines are stable components of the *B. tabaci* metabolome and can be reproducibly quantified in a relative manner, providing a robust analytical foundation for subsequent analyses of polyamine dynamics under controlled experimental conditions.

**Figure 3:**
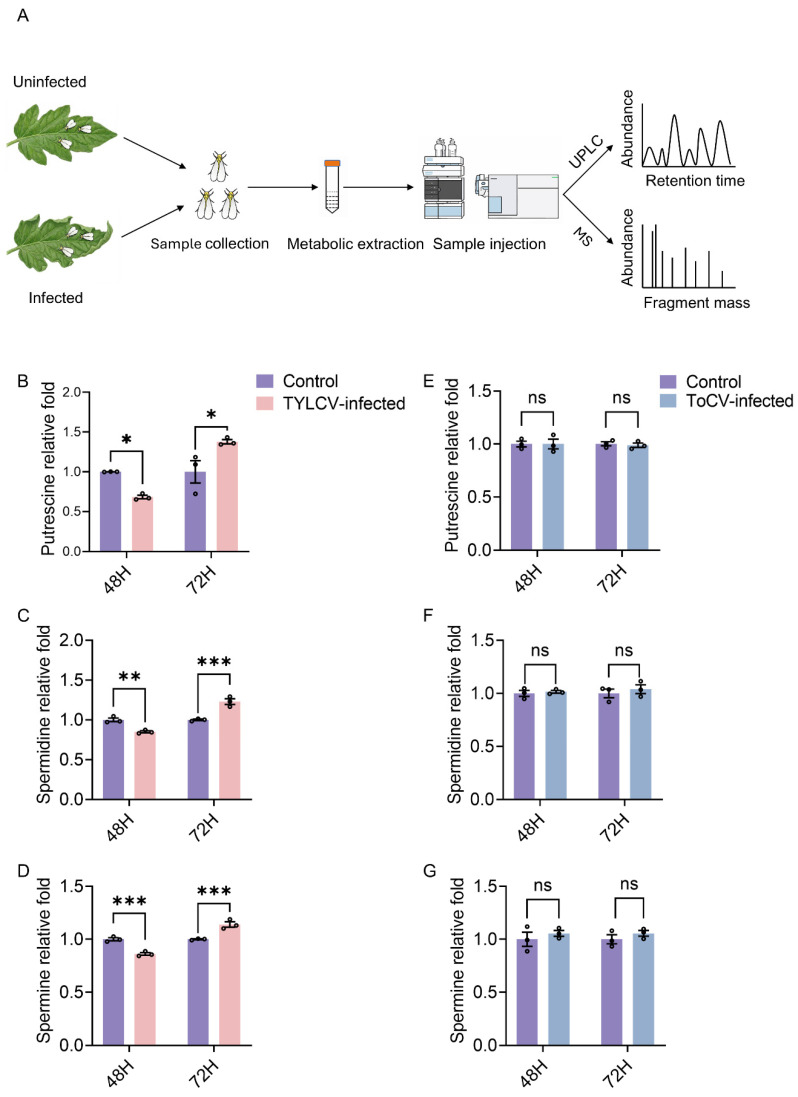
Polyamine levels in *B. tabaci* following TYLCV or ToCV infection. (**A**) Procedure for UPLC-QTOF/MS analysis of polyamine from whiteflies. Whiteflies were frozen and ground into powder. The metabolites were extracted with 10% perchloric acid (PCA) and were analyzed using the ACQUITY UPLC I-Class/Xevo G2-XS QTOF system. (**B**–**D**) Polyamine levels in *B. tabaci* at 48 h and 72 h after acquisition feeding on TYLCV-infected tomato plants, as determined by UPLC-MS/MS. (**B**) Putrescine; (**C**) spermidine; (**D**) spermine. (**E**–**G**) Polyamine levels in ToCV-infected whiteflies at 48 h and 72 h. Data were presented as mean ± SEM from three independent biological replicates, with 10 mg whiteflies per replicate. Statistical significance was determined by two-way ANOVA followed by Dunnett’s multiple comparison test. ns, not significant; * *p* < 0.05; ** *p* < 0.01; *** *p* < 0.001.

To test whether TYLCV acquisition is accompanied by polyamine remodeling, putrescine (Put), spermidine (Spd), and spermine (Spm) were quantified in whole body extracts using the polyamine detection method developed (Figure 3A). Polyamine levels were quantified in whole body extracts of *B. tabaci* at 48 h and 72 h after TYLCV acquisition. In TYLCV-infected whiteflies, putrescine, spermidine, and spermine levels decreased at 48 h compared to the control group but increased at 72 h, showing a time-dependent modulation (Figure 3B–D). By contrast, no significant changes in polyamine levels were observed in ToCV-infected whiteflies, suggesting that the changes in polyamine levels were specific to TYLCV infection (Figure 3E–G). These findings indicate the virus induced metabolic shifts in polyamine metabolism, reflecting the different mechanisms by which these two viruses interact with the vector, with TYLCV’s ability to replicate and persist in the whitefly driving the observed polyamine remodeling. 

### 3.3. Polyamine Abundance Positively Associates with TYLCV DNA Accumulation Across Cohorts

**Figure 4:**
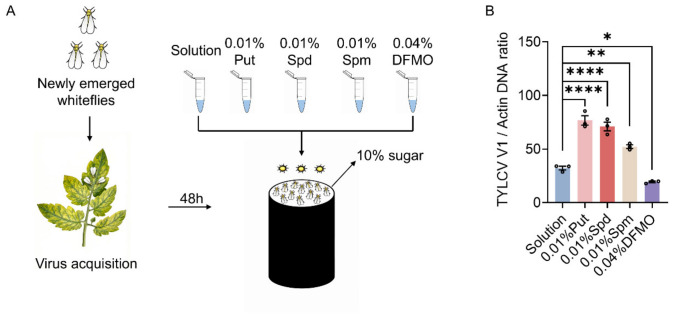
Effect of exogenous polyamine and DFMO supplementation on *B. tabaci* TYLCV accumulation. (**A**) Emerged whiteflies were allowed to acquire TYLCV from infected tomato plants for 48 h before supplementation with 0.01% putrescine (Put), spermidine (Spd), spermine (Spm), or 0.04% DFMO in 10% sucrose solution. (**B**) Viral load was quantified by qPCR and normalized to the host *actin* gene. Data were presented as mean ± SEM from three independent biological replicates with 30 whiteflies per replicate. Statistical significance was determined by one-way ANOVA followed by Dunnett’s multiple comparison test. ns, not significant; * *p* < 0.05; ** *p* < 0.01; **** *p* < 0.0001.

To assess whether polyamine abundance influences viral accumulation within whiteflies, whiteflies fed on TYLCV-infected tomato for 48 h and then were treated with different polyamines (putrescine, spermidine, or spermine), and a polyamine synthesis inhibitor (DFMO) (Figure 4A). TYLCV DNA levels were quantified by qPCR, normalized to the host *β*-*actin* gene. Supplemented with putrescine, spermidine, or spermine significantly increased whiteflies’ TYLCV DNA abundance (Figure 4B), indicating that elevated polyamine availability favors viral accumulation. However, supplementation with DFMO significantly reduced TYLCV DNA levels, demonstrating a clear inhibitory effect on virus accumulation. Consistent with these observations, time-course analyses revealed that TYLCV DNA levels increased progressively during prolonged acquisition, reaching maximal levels at later time points that coincided with elevated endogenous polyamine abundance (Figure 1B and Figure 4B). These results demonstrate that polyamine availability is positively associated with the extent of TYLCV accumulation during sustained infection in whiteflies.

### 3.4. RNAi-Mediated BtODC Knockdown Suppresses TYLCV DNA Accumulation

**Figure 5:**
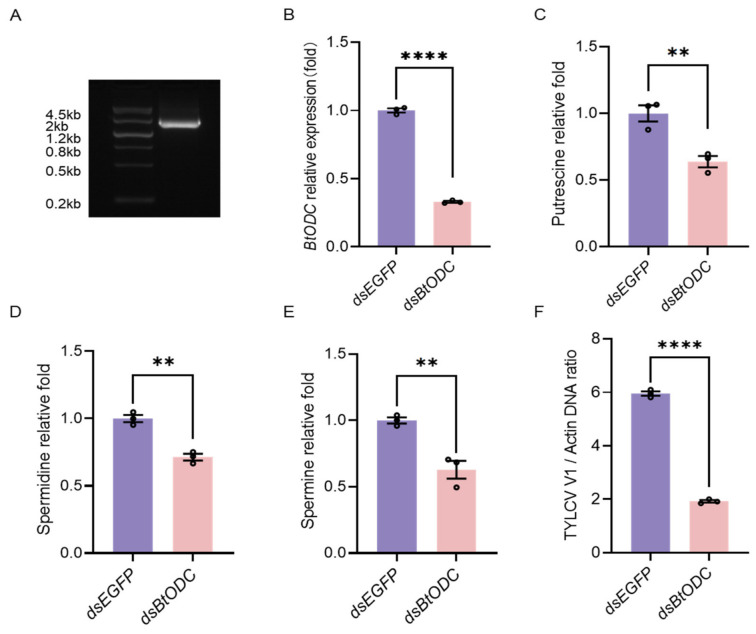
*BtODC* knockdown reduces TYLCV DNA accumulation in *B. tabaci.* (**A**) PCR-based verification of the cloned *BtODC* fragment. (**B**) Relative expression level of *BtODC* after RNAi-mediated knockdown. (**C**–**E**) Endogenous putrescine (**C**), spermidine (**D**), and spermine (**E**) levels in *B. tabaci* after *BtODC* knockdown. (**F**) Effect of *BtODC* knockdown on TYLCV DNA accumulation in whiteflies. Data were shown as mean ± SEM. Statistical significance was determined by one-way ANOVA followed by Tukey’s multiple comparison test; ** *p* < 0.01; **** *p* < 0.0001.

To further investigate whether endogenous polyamine biosynthesis contributes to TYLCV accumulation, the *BtODC* gene was identified from the *B. tabaci* genome through BLAST sequence analysis. *BtODC* encodes ornithine decarboxylase, the rate-limiting enzyme in putrescine biosynthesis. A fragment of the *BtODC* gene was subsequently cloned for further analysis (Figure 5A), and double-stranded RNA (dsRNA) targeting *BtODC* was designed for RNA interference experiments.

RT-qPCR analysis (Figure 5B) showed that, compared with the *dsEGFP* treated control group, treatment with *dsBtODC* significantly reduced the transcript abundance of *BtODC*, indicating effective RNAi-mediated gene silencing. Further quantification of endogenous putrescine, spermidine, and spermine levels in *B. tabaci* revealed that all three polyamines were significantly reduced following *BtODC* knockdown (Figure 5C–E), suggesting that silencing of *BtODC* effectively suppressed endogenous polyamine biosynthesis. Meanwhile, TYLCV DNA levels in *BtODC*-silenced whiteflies were reduced by approximately 60% compared with the control group (Figure 5F). This result was consistent with the inhibitory effect observed following DFMO treatment and further supports the involvement of endogenous polyamine biosynthesis in TYLCV accumulation within the whitefly vector.

### 3.5. Exogenous Polyamines Suppress the Expression of Immune and MAPK-Related Genes in TYLCV-Infected B. tabaci

**Figure 6:**
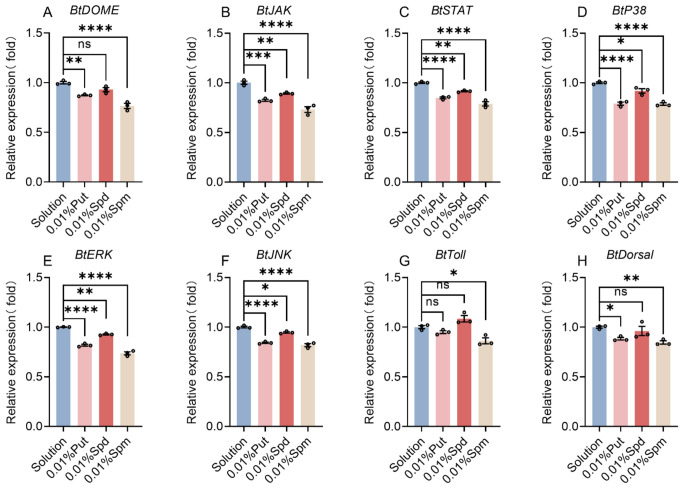
Effects of exogenous polyamine feeding on immune and MAPK signaling-related gene expression in TYLCV-infected *B. tabaci*. Adult whiteflies fed on TYLCV-infected tomato plants for 48 h and then were supplemented with putrescine (Put), spermidine (Spd), spermine (Spm), or solvent control for an additional 48 h. Transcript levels of *BtDOME* (**A**), *BtJAK* (**B**), *BtSTAT* (**C**), *BtP38* (**D**), *BtERK* (**E**), *BtJNK* (**F**), *BtToll* (**G**), and *BtDorsal* (**H**) were quantified by RT-qPCR and normalized to the reference gene. Data were presented as mean ± SEM from three independent biological replicates. Statistical significance was determined by one-way ANOVA, followed by Dunnett’s multiple comparisons. ns, not significant; * *p* < 0.05; ** *p* < 0.01; *** *p* < 0.001; **** *p* < 0.0001.

To investigate whether polyamines modulate the host immune response, we analyzed the transcriptional changes of key immune genes in TYLCV-infected *B. tabaci* following treatment with 0.01% exogenous putrescine, spermidine, and spermine (Figure 6). Specifically, putrescine and spermine significantly downregulated all key components of the JAK/STAT pathway, including *BtDOME*, *BtJAK*, and *BtSTAT*. However, spermidine exhibited selective inhibition, significantly reducing *BtJAK* and *BtSTAT* levels while leaving *BtDOME* unaffected. A similar suppressive pattern was observed in the MAPK signaling pathway, where *BtP38*, *BtERK*, and *BtJNK* were inhibited by putrescine, spermidine, and spermine. In the Toll signaling pathway, spermine exerted the strongest effect by downregulating both *BtToll* and *BtDorsal*, whereas putrescine only suppressed the downstream factor *BtDorsal*. Collectively, these findings indicate that elevated polyamine levels may promote TYLCV load, further suppressing the host immune system and MAPK pathway.

Experiments conducted in non-viruliferous whiteflies showed that exogenous polyamine supplementation also led to a downward trend in the expression of some immune and MAPK-associated genes (Figure S1). However, the transcriptional changes were more pronounced under TYLCV infection conditions, suggesting that the observed responses are likely influenced by the combined effects of altered polyamine availability, TYLCV infection status, and increased TYLCV DNA accumulation. Elevated polyamine levels may therefore contribute to the attenuation of immune and MAPK-associated transcriptional responses in TYLCV-infected *B. tabaci*, thereby potentially facilitating viral accumulation within the whitefly vector.

## 4. Discussion

Polyamines are vital for viral replication, influencing multiple stages of the viral life cycle, from genome stabilization to the promotion of viral protein synthesis [35]. In HSV-1, both spermidine and spermine are found within the virus, with spermidine located in the envelope and capsid, a distribution likely essential for maintaining viral genome stability and packaging efficiency [36]. Additionally, polyamines enhance viral enzyme activities that are crucial for genome replication and protein translation. In bacteriophages like T4, spermidine plays a significant role in aiding the packaging of viral nucleic acids [35]. In coronaviruses, polyamine depletion significantly reduces viral attachment and entry, demonstrating that polyamines play a significant role in enhancing viral attachment and entry into host cells [37].

This study highlights the time-resolved quantification of polyamines in *B. tabaci* following TYLCV acquisition, revealing a biphasic pattern. During the early phase (48 hpi), the levels of putrescine, spermidine, and spermine declined significantly, whereas at later stages (72 hpi), all three polyamines accumulated to levels well above those in uninfected controls. This dynamic pattern suggests a regulated metabolic interplay between the virus and its vector. The early phase reduction in polyamine levels could be a host-driven metabolic strategy to restrict viral accumulation. This strategy, known as ‘polyamine depletion,’ limits the availability of critical metabolites required for viral propagation. Zika virus (ZIKV), Chikungunya virus (CHIKV), Herpes simplex virus (HSV), Human cytomegalovirus (HCMV), and Influenza A virus (IAV) rely on polyamines, where polyamines promote these viral genomes’ replication and translation. Host-induced inhibition of polyamine synthesis limits the replication and propagation of these viruses [32,38,39,40]. In the case of *B. tabaci* and TYLCV interaction, the early decrease in polyamine levels may act as an intrinsic metabolic defense within the vector, restricting the virus’s ability to successfully establish itself during the early stages of infection.

As infection progressed, however, TYLCV appeared to overcome this constraint, driving a marked accumulation of polyamines at later time points. Such metabolic reprogramming is a well-established strategy employed by viruses to create a proviral intracellular environment [20]. The late-stage elevation of polyamines observed here coincided temporally with rapid viral DNA accumulation, supporting a functional link between host polyamine pools and TYLCV persistence in the vector. Notably, this biphasic pattern was specific to TYLCV and was not detected in whiteflies infected with the non-circulative virus ToCV, although ToCV levels also increased progressively with acquisition time [41]. ToCV is transmitted in a semi-persistent, non-circulative manner. Therefore, the increase in ToCV levels is more likely attributable to virus acquisition by the whitefly vector and the binding or retention of virions at mouthpart or foregut-associated sites. Importantly, despite the successful acquisition of ToCV, no corresponding biphasic remodeling of putrescine, spermidine, or spermine was observed, suggesting that the polyamine remodeling in TYLCV-infected whiteflies is not merely caused by virus accumulation itself. The results suggest that DNA viruses such as begomoviruses may have evolved specialized mechanisms to manipulate polyamine metabolism in their insect vectors. Both Rice yellow stunt virus (RYSV) and Rice stripe mosaic virus (RMSV), transmitted by leafhoppers in a persistent propagative manner, influence polyamine metabolism to promote viral replication [33,34]. This shared infection pattern with TYLCV suggests that TYLCV also manipulates polyamine pathways in a similar way to ensure its persistence in insect vectors.

Previous studies have shown that TYLCV infection significantly alters the composition of free amino acids in tomato phloem sap, adult whiteflies, and honeydew [42], while polyamine biosynthesis is highly dependent on amino acid metabolism. Specifically, glutamate and proline metabolism may provide precursor substrates for ornithine synthesis in *B. tabaci* [43], and ornithine serves as an important initiating molecule for polyamine biosynthesis. In addition, methionine-derived S-adenosylmethionine provides aminopropyl groups for the synthesis of spermidine and spermine. Therefore, TYLCV-induced alterations in amino acid metabolism may provide substrate level or regulatory support for the polyamine metabolic remodeling observed in this study.

Functional perturbation experiments further support the involvement of polyamines in regulating viral DNA accumulation. Pharmacological inhibition of polyamine biosynthesis using DFMO significantly reduced TYLCV DNA levels, whereas exogenous supplementation with putrescine, spermidine, or spermine enhanced viral accumulation. Furthermore, RNAi-mediated silencing of *BtODC*, which encodes the rate-limiting enzyme involved in polyamine biosynthesis in *B. tabaci*, significantly reduced endogenous polyamine levels in whiteflies and concomitantly decreased TYLCV DNA accumulation. These results indicate that endogenous polyamine biosynthesis in *B. tabaci* contributes to TYLCV accumulation within the whitefly vector. These reciprocal effects indicate that polyamine availability acts as a rate modulating factor for TYLCV persistence within the vector. During long distance transport in the insect body, viral particles face mechanical stress and enzymatic degradation, especially in the hemolymph and salivary glands [12,13]. Given the well-established biochemical properties of polyamines, including their ability to neutralize negative charges and condense nucleic acids [29,36,44], we hypothesize that elevated polyamine levels may stabilize viral ssDNA and facilitate genome packaging, thereby enhancing TYLCV integrity and persistence during circulative transport, further promoting viral accumulation.

Polyamine supplementation results in the suppression of key immune and MAPK pathway genes, including those in the JAK/STAT, Toll, and MAPK signaling pathways in TYLCV-infected *B. tabaci*. These pathways are essential for immune signaling and stress responses. Persistent, circulative transmission of begomoviruses requires the coordinated navigation of multiple vector barriers, together with the capacity to evade or tolerate host immune surveillance [12,13,14]. Previous studies have demonstrated that TYLCV interferes with the JAK/STAT immune signaling pathway in *B. tabaci* [22], and therefore, we hypothesize that exogenous polyamine supplementation enhances viral load, which further suppresses immune signaling pathways such as JAK/STAT, Toll, and MAPK, thereby weakening the host’s antiviral immune responses. In this model, polyamines act as facilitators of maintaining viral stability and promoting immune evasion. In HSV-1-infected mouse tissues, elevated polyamine levels regulate the transition from B-form DNA to Z-form DNA, thereby reducing the binding affinity of DNA to cGAS. This attenuation of cGAS activation suppresses the antiviral immune response, leading to decreased type I interferon production, which in turn enhances viral replication and immune evasion [45]. Although a similar trend was also observed in non-viruliferous whiteflies following polyamine supplementation, the transcriptional changes were substantially weaker than those detected in TYLCV-infected whiteflies, suggesting that the observed attenuation of immune and MAPK associated transcriptional responses is likely caused by the combined effects of multiple factors.

**Figure 7:**
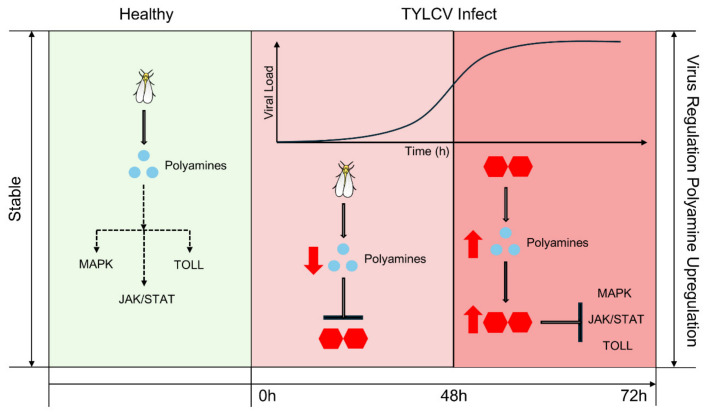
This model illustrates the dynamic regulation of polyamines and their effects on the immune and MAPK signal pathways in *B. tabaci* during TYLCV infection. In the healthy state (green area), polyamines remain stable. During the initial 48 h of infection (pink area), the whitefly actively downregulates polyamine levels to prevent excessive viral accumulation. However, as the infection progresses (48–72 h; light red area), the virus overcomes this resistance, upregulating polyamines to enhance its accumulation and suppress the immune system. This dynamic regulation of polyamines helps virus persistence in whitefly. The direct impact of polyamines on immune and MAPK signal pathways in whitefly remains an open question (dotted line). Red arrows represent regulatory changes (upregulation or downregulation), while black blunt-ended lines indicate inhibition.

Collectively, our results support a model in which TYLCV dynamically reprograms polyamine metabolism to orchestrate a transition from early metabolic restriction to late metabolic permissiveness (Figure 7). This biphasic strategy appears to integrate structural stabilization of viral DNA with modulation of host immune signaling, jointly enhancing viral accumulation. Such coordinated manipulation underscores the sophistication of begomovirus–vector interactions and highlights polyamine metabolism as a regulatory factor at the interface of viral persistence, vector physiology, and disease spread. However, whether this regulatory mechanism is conserved among other cryptic species of *B. tabaci* requires further investigation.

## Data Availability

Any further data may be obtained from the authors upon reasonable request.

## Supplementary Materials

The following supporting information can be downloaded at: https://www.mdpi.com/article/10.3390/molecules31111835/s1. Table S1. Primers used in this study; Excel S1: Data Processing Summary Table for the Manuscript.
