# Supplementary material for: Tomato Yellow Leaf Curl Virus Reprograms Polyamine Metabolism in Bemisia tabaci MED to Enhance Viral DNA Accumulation
Source: Molecules. 2026 May 26;31(11):1835. doi: 10.3390/molecules31111835 (PMC13258564; doi:10.3390/molecules31111835)
Supplement: Supplementary file 1 [file molecules-31-01835-s001.zip › Primer Table.pdf]

Table S1. Primers used in this study.

Primer	Sequences(5'-3')	Purpose
Q-F	CTTGGTAACTCTTCTGTAGATGTGTGTT	B/Q Whitefly identification
Q-R	CCTTCCCGCAGAAGAAATTTTGTTC	
β-actin-F	TCTTCCAGCCATCCTTCTTG	qRT-PCR for β-actin
β-actin-R	CGgtGATTTCCTTCTGCATT	
qTYLCV-F	GAAGCGACCAGGCGATATAA	qRT-PCR for TYLCV total DNA
qTYLCV-R	GGAACATCAGGGCTTCGATA	
qBtDOME -F	CAGTGCGAGGGAATTTCTACA	qRT-PCR for JAK/STAT pathway
qBtDOME -R	ACACCATACCACTGCTTCCA	
qBtJAK -F	TTGATGAGCTTGCACCAAAT	
qBtJAK -R	TTCCTTCAAAGCACATCCTG	
qBtSTAT -F	CCATTTCACTGTTGGTGGAG	
qBtSTAT -R	AACGGTATACGCCCAAGTTC	
qBtP38-F	GAACGCCGTCGGAGGATACTT	qRT-PCR for MAPK signal pathway
qBtP38-R	TTGGCTCCTTTGAACACTTGC	
qBtERK-F	AGATTATTTCCTTCAGCCGATGC	
qBtERK-R	GGGCAAGGGCATCTTCAACTAC	
qBtJNK-F	TGTTGAGCAGTGGAAAGAGC	
qBtJNK-R	TTCGATAGACGGACTCGTTG	
qBtToll -F	TGGAATCAACACCTCCAGCTA	qRT-PCR for TOLL pathway
qBtToll -R	CCTTGAGTATTTCAATCCACAAATA	
qDorsal -F	AAGGCTCATCCGCACAAAC	
qDorsal -R	AATTCGGGAAGCTGGCAGTC	
